# The Early Stage Adjacent Disc Degeneration after Percutaneous Vertebroplasty and Kyphoplasty in The Treatment of Osteoporotic VCFs

**DOI:** 10.1371/journal.pone.0046323

**Published:** 2012-10-08

**Authors:** Jun Qian, Huilin Yang, Juehua Jing, Hong Zhao, Li Ni, Dasheng Tian, Zhengfei Wang

**Affiliations:** 1 Department of Orthopaedics, The First Affiliated Hospital of Soochow University, Suzhou, Jiangsu Province, People's Republic of China; 2 Department of Orthopaedics, The Second Hospital of Anhui Medical University, Hefei, Anhui Province, People's Republic of China; 3 Department of Radiology, The Second Hospital of Anhui Medical University, Hefei, Anhui Province, People's Republic of China; Georgia Health Sciences University, United States of America

## Abstract

**Background:**

The purpose of this paper is to determine the early incidence of disc de- generation adjacent to the vertebral body of osteoporotic fracture treated with percutaneous vertebroplasty or balloon kyphoplasty and whether adjacent disc degeneration is accelerated by this two procedures.

**Methods:**

182 patients with painful vertebral compression fractures were treated. A total of 97 patients were enrolled in this prospective study. 97 patients with a mean age of 65.3 years were classified into control group and surgical treatment group of non-random. 35 patients were in contol group and 62 patients who were performed percutaneous vertebroplasty or balloon kyphoplasty in treatment group. X-ray and Magnetic resonance imaging were done at the first and final visit. The grade of disc degeneration above the fractured vertebral was confirmed by evaluation of bony oedema in the fat suppressed sequences and T2-weighted image of magnetic resonance imaging. The height of degenerative disc was measured on X-ray film.

**Results:**

All patients were followed up two years after the first visit and the follow-up rate was 90.7% (88/97). The incidence of degeneration of adjacent disc above the fractured vertebral was 29.0% (9/31) in control group and 52.6% (30/57) in treatment group. It presented a statistically significant difference between two groups about the incidence of adjacent disc degeneration (*P* = 0.033). The percentage of adjacent disc height reduction in control group was 13.5% and 17.6% in treatment group. Statistically significant difference of VAS score and ODI was not found between the first evaluation postoperatively and the final follow-up in treatment group (*P*>0.05).

**Conclusions:**

Disc degeneration adjacent to the fractured vertebral is accelerated by VP and BK procedures in the early stage, but clinical outcomes has not been weakened even in the presence of accelerated disc degeneration.

## Introduction

Osteoporosis is a systemic disease that compromises bone strength and predisposes patients to an increased risk of fractures [1]. It is estimated to afflict 200 million women worldwide and reported a total of 700,000 vertebral fractures in USA each year [2]. Vertebral compression fractures (VCFs) constitute a major health care problem worldwide, not only because of their high incidence but also due to their negative consequences on the patient's health-related quality of life and the high costs to the health care system [3], [4], [5]. Vertebral compression fractures can result in chronic back pain, kyphotic deformity and disability. The conventional treatments include medications, bed rest and bracing. But they are only partially effective for people suffering from osteoporotic VCFs. Previous studies have found that conservative treatments of symptomatic VCFs often fail to improve pain and mobility particularly in cases of chronic pain related to kyphotic deformity [6]. Therefore, VCFs were gradualy treated with minimally invasive approaches such as percutaneous vertebroplasty (VP) or percutaneous balloon kyphoplasty (BK) in the past two decades [7].

VP was first described in 1987 by Galibert et al [8]. VP involves the percutaneous injection of bone cement, such as polymethylmethacrylate (PMMA), directly into the cancellous bone of a vertebral body. This technique of VP was successfully used by Galibert to treat seven patients who had painful vertebral angiomas. Since then, the use of vertebroplasty has become popular for alleviating pain associated with a vertebral compression fracture and preventing the further loss of vertebral height or the progression of a kyphotic deformity [9]. However, This technique has some shortcomings including complication of cement extravasation and failure to correct the kyphotic deformity caused by vertebral compression fractures [10], [11]. In some cases, cement leakage into the epidural can result in paralysis due to spinal cord compression [12].

BK was introduced in 1998 as an improvement of VP [13]. BK was introduced by Kyphon to describe balloon-assisted vertebroplasty. In comparison with VP, a deflated balloon is inserted into the vertebral body through the pedicle and inflated to restore the height of a collapsed vertebral body and create a cavity inside. The balloon is then deflated and withdrawn. After removal of the balloons, PMMA is injected into the cavity at a proper velocity. Theoretically, the cavity made by the balloon allow the cement to be injected under low manual pressure and minimize the cement leakage [9], [14]. A unipedicular approach is often sufficient in vertebroplasty, kyphoplasty is often performed bipedicularly [15]. BK is a minimally invasive procedure aiming to palliate back pain, stabilize the fracture, restore lost vertebral body height and correct kyphotic deformity [16].

Although VP and BK compared with conservative treatments have shown obvious therapeutic effects on osteoporotic vertebral compression fractures, especially in relieving back pain. There are still some concerns about the safty and impact on adjacent segment resulting from VP and BK procedures [17], [18], [19]. Since the inception of these procedures, many previous literatures evaluated the impact on the adjacent segment vertebral after the two procedures. However, there is not a published literature which evaluates the degeneration of adajecent disc after VP and BK procedures in the treatment of VCFs until now. The purpose of this study was to investigate the incidence of adjacent disc degeneration after the two procedures in the early stage and to determine whether the two procedures can accelerate the adjacent disc degeneration above the treated vertebral.

## Patients and Methods

We have received the agreements of all patients.

1. We have obtained the approval from ethics committee of our hospital.
2. We did not conduct our research outside of our country of residence.
3. All patients have provided their written informed consent to participate in this study.
4. The ethics committees of our hospital have approved this consent procedure.

### Patients

A prospective study was conducted in this paper. Over a 3.9-year period, 182 consecutive patients with painful lumbar VCFs were treated in two hospitals. All patients had a similar history of slipping or wrestling and underwent X-ray film, computed tomography (CT) and magnetic resonance imaging (MRI) scan. The diagnosis of osteoporotic VCF was established by the clinical examination and radiographic evaluation. A total of 97 patients were enrolled in this prospective study after the exclusion of 85 patients who should be treated with an open surgery or sever degeneration of adjacent discs had occurred at the first visit. All objective patients (72 females and 25 males) with a mean age of 65.3 years (range, 53–78 years) were classified into control group (conservative group) and surgical treatment group of non-random. 35 patients were treated conservatively in contol group and 62 patients were performed VP (9 patients) or BK (53 patients) in surgical treatment group. All patients in two groups had a similar symptoms of only back pain, grade and level of slip and life style. Demographic characteristics of patients in each group were summarized in Table 1.

**Table 1:** Comparison of demographic and baseline characteristics between patients treated with vertebroplasty (VP) and patients treated with balloon kyphoplasty (BK).

Characteristics	Control group	Treatment group	P value
	(n = 35)	(n = 62)	
Age ( 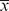 ±s, years)	63.7±8.3	66.2±10.8	0.239
Gender			0.256
Women	28	43	
Men	7	19	
Level			0.573
L1	24	38	
L2	6	13	
L3	4	8	
L4	1	3	
Smokers	5	7	0.667
Weight ( 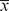 ±s, kg)	63.2±10.5	67.1±11.8	0.097
Mean adjacent disc height ( 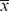 ±s, mm) (above the fractured vertebral,preoperative)	11. 2±2.3	11. 0±1.9	0.650
Average height loss ( 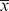 ±s, mm) (the fractured vertebral,preoperative)	19.2±7.6	17.9±8.2	0.305
Preoperative VAS Score	7.1±1.8	7.6±2.2	0.230
Preoperative ODI	65.7±15.1	68.1±16.2	0.466

### Inclusion and Exclusion

All patients in two groups should be diagnosed as painful lumbar VCFs and had only back pain. All VCFs should be suitable for treated with VP or BK techniques. The patients who need an open surgery or severe degeneration of adjacent discs had occurred at the first visit were excluded. Finally, 97 patients were enrolled in this prospective study. 35 of 97 patients were taken a conservative treatment, the reasons for refusing surgery including lack of the cost of VP or BK in 19 patients, fear of the complications of the surgery in 12 patients and showing contraindications to surgery and anesthesia in 4 patients. All patients treated conservatively were integrated the control group. We constructed the surgical treatment group with 62 underwent VP and BK procedures, VP group 9 patients and BK group 53 patients. All VCFs treated by VP or BK were located in the lumbar spine.

### Control group

In this group composed of 28 women and 7 men, mean age was 64.7 years (range, 53–73 years). Out of 35 VCFs, 24 (68.6%) located in L1, 6 (17.1%) located in L2, 4 (11.4%) located in L3 and 1 (2.9%) located in L4. These patients were treated with similar methods, including a short period of bedrest, avoiding excessive bending and other prolonged activities. Oral or parenteral analgesics were administered for pain control and vitamin D and calcium had be used for improving the quality of bone. Muscle exercises, external backbraces, and physical therapy modalities were also be used during the process of treatment [20], [21].

### Treatment group

62 patients were enrolled in this group, 43 women and 19 men. Mean age was 66.2 years (range, 56–78 years). 38 (61.3%) located in L1, 13 (21.0%) located L2, 8 (12.9%) located in L3 and 3 (4.8%) located in L4. 9 patients were underwent VP at the early stage of that 3.9-year period. VP procedures were performed under general anesthesia using an 11 gauge trochar needle to cannulate the pedicle with the guidance of fluoroscopy. Needles were advanced to the anterior first third of the vertebral body under C-arm fluoroscopic aid. The liquid polymermethylmethacrylate (PMMA) was mixed with the powder to a dough-like texture. Under image control, the bone cement was injected alternatively through the left or right needles by using 1.5-ml syringes. 9 patients were all completed by uni-pedicular approach. Mean volume of 2.2 mLs (range 1.5–3 mLs) cement was injected into the fractured vertebral body.

BK technique was used in 53 patients. BK were also performed under general anesthesia. Patients were placed in a prone position. A 0.5 cm skin incision positioned approximately 1–2 cm lateral to the appropriate pedicle was made around the fractured vertebral pedicle under C-arm fluoroscopic guidance. Two 11 gage trochar needles were inserted into the pedicle percutaneously. Two 1.5 mm diameter guide pins were inserted through the trochar needles, and then two inflatable bone tamps were inserted into the fractured vertebral bodies. The balloons were dilated simultaneously under C-arm fluoroscopic monitoring. Ballooning pressures did not exceed 200 psi, and a balloon cavity was made in the vertebral body with 3–4 mLs cavity. Then, bone cement containing 1.5 mLs of PMMA was injected through the filler. From both sides, Mean volume of 3.9 mLs (range 3–5.5 mLs) cement was injected into the fractured vertebral body in most patients.

Following VP or BP procedures, every patient was monitored in patient's ward where they lied supine for more than 6 hours. This allowed both time for the cement to set and the patients to recovery from sedation. Patients could be discharged from hospital at the next day after operation and advised to take some medications such as vitamin D and calcium.

### Radiological measurements

All patients were followed up until two years after the discharge from hospital. The first examinations were conducted during the first visit to hospital or before the surgery, while the follow-up visits with X-ray films were carried out on the first day, 3 months, 1 year, and 2 year after surgery. But MRI scan were performed only at the first and last visits in order to save costs. The visits at the 3 months and 1 year postoperatively were not considered in this analysis.

Radiological criteria were used to define degneration of the adjacent disc. The degree of lumbar disc degeneration was categorized into five grades by one blinded spine surgeon according to the grading system proposed by Pfirrmann et al [22] (Table 2). This system evaluates intervertebral disc structure, distinction of the nucleus and anulus, signal intensity of the disc, and disc height on T2-weighted midsagittal images. The reliability of this grading system has been tested and was shown to achieve excellent agreement in some previous reports [22], [23]. The adjacent disc was considered to be involved when, on standard lateral X-ray films, adjacent disc height reduction (DHR) amounts approximately 20%. This need to be interpreted as disc degeneration [24], [25]. The grade of disc degeneration adjacent to the fractured vertebral body was confirmed by the evaluation of the bony oedema in the fat suppressed sequences (STIR) and T2-weighted image of the magnetic resonance imaging in the sagittal plane and the height of degenerative disc was measured on the X-ray films. Adjacent anterior disc height and posterior disc height were measured on the lateral radiographs. Then, mean height of adjacent disc was calculated by the mean value of anterior height and posterior height. The imaging datas of all patients were measured by a surgeon and a radiologist respectively.

**Table 2:** Disc degeneration grading according to Pfirrmann *et al*.

Grade	Structure	Distinction of	Signal Intensity	Intervertebral
		Nucleus and Anulus		Disc Height
I	Homogeneous bright white	Clear	Hyperintense,isointense to cerebrospinal fluid	Normal
II	Heterogeneous with or without horizontal bands	Clear	Hyperintense,isointense to cerebrospinal fluid	Normal
III	Heterogeneous, gray	Unclear	Intermediate	Normal to lightly decreased
IV	Heterogeneous gray to black	Lost	Intermediateto hypointense	Normal to oderately decreased
V	Heterogeneous, black	Lost	Hypointense	Collapsed disc

### Clinical examination

Patients were evaluated for pain by using the visual analog scale (VAS) score. Scores range from 0 (no pain) to 10 (extreme pain) [26]. Functional Disability was measured by the oswestry disability index (ODI). The ODI is a validated disease specific instrument for assessment of spinal disorders consist's of a 10-item ordinal scale with 6 response alternatives for each item [27]. The total score ranges from 0 to 100, where 100 is worst disability. Back pain and functional disability of all patients were assessed according to VAS score and ODI at the first visit and every follow-up.

### Statistical Analysis

Continuous data were presented as the mean ± standard deviation, unless otherwise specified. A t test was used to analyze different characteristics of patients between two groups and the Chis quared test was used to test the incidence of adjacent disc degeneration. P<0.05 was considered as statistically significant. Statistical analyses were performed independently by a non-clinical research assistant to ensure objectivity, using SPSS Version 13.0 software.

## Results

A total of 88 patients were followed up and the follow-up rate was 90.7%. The average follow-up time was two years. There was no significant difference in age, gender, body weight and the number of smokers between control group and surgical treatment group. Additionally, a statistically significant difference could not be confirmed with regard to the level of VCFs, average height loss of the fractured vertebral body, VAS score and ODI preoperatively between the two groups. P values of all parameters between the two groups were displayed in the Table 1. Mean preoperative height of ajacent disc in the control group was 11.2±2.3 (mm), presenting no statistical difference with the surgical treatment group 10.7±1.9 (mm) (p = 0.306).

According to the degeneration grading system proposed by Pfirrmann et al (Table 2), 44.3% (39/88) of all patients were found to have an adjacent disc degeneration through MRI scan, 29.0% (9/31) patients in control group and 52.6% (30/57) patients in trentment group. Of the surgical treatment group, the incidence of adjacent disc degeneration was 66.7% (6/9) patients in VP group and 45.3% (24/53) patients in BK group. Obviously, patients treated with VP and BK procedures had a statistically significant chance (P = 0.033) of accelerating adjacent disc degeneration comparied with the control group. The number of patients performed VP had a small sample size, so we didn't carry out a statistics analysis between VP and BK groups.

**Figure 1:**
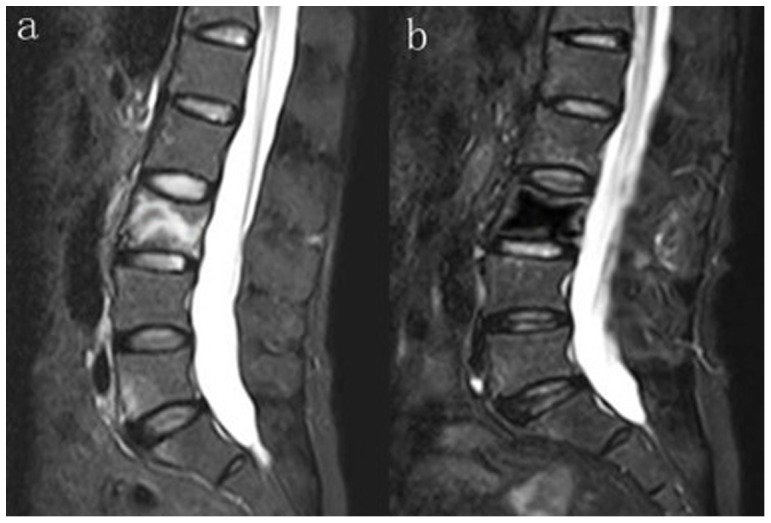
T2-weighted image of MRI in the sagittal plane. (a) Preoperative photograph of VCF of L3 (b) Postoperative photograph of Grade II of degenerative disc above the treated vertebral.

**Figure 2:**
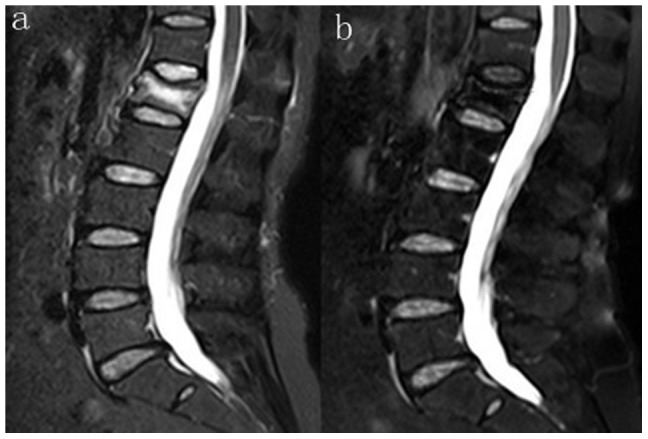
T2-weighted image of MRI in the sagittal plane. (a) Preoperative photograph of VCF of L1 (b) Postoperative photograph of Grade III of degenerative disc above the treated vertebral.

**Figure 3:**
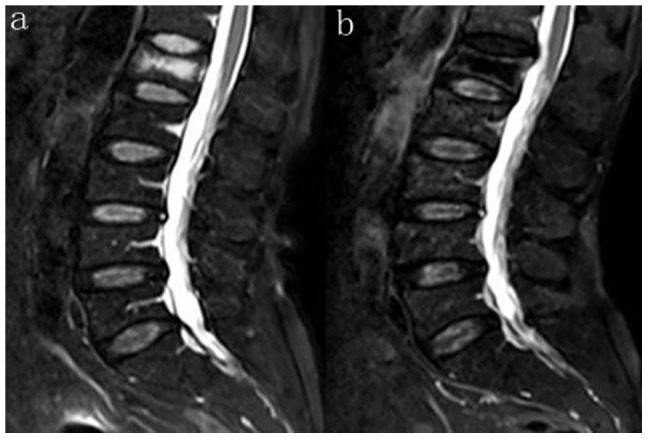
T2-weighted image of MRI in the sagittal plane. (a) Preoperative photograph of VCF of L1 (b) Postoperative photograph of Grade IV of degenerative disc above the treated vertebral.

9 patients in control group had adjacent disc degeneration, 3 patient grade II, 5 patients grade III, 1 patients grade IV. The level of adjacent disc degeneration was also observed. 6 degenerative dicses located in T12/L1, 3 degenerative dicses in L1/2. In treatment group, 7 patients grade II (Figure 1), 18 patients grade III (Figure 2), 5 patients grade IV (Figure 3), and 16 degeneretion dicses situated at T12/L1, 13 degeneretion dicses at L1/2, 1 degeneretion dicses at L2/3. The incidence of degeneration in different lumbar level displayed a nonsignificant difference between two groups (P>0.05). (Table 3)

**Table 3:** Number of patients with adjacent disc degeneration in two groups.

Grade	Control group (n = 31)	Treatment group (n = 57)
I	0	0
II	3	7
III	5	18
IV	1	5
V	0	0

*P* = 0.033.

The percentage of DHR of the adjacent disc in control group was 13.5% and 17.6% in treatment group. The percentage of DHR of the adjacent disc presented a nonsignificant difference between two groups (P = 0.553), and the percentage of each group was less than 20%, according to the X-ray radiographs criteria of disc degeneration, adjacent disc degeneration would not be confirmed in two groups. However, a higher proportion (39/88) of adjacent disc degeneration appeared in MRI scan according to the degeneration grading system proposed by Pfirrmann et al. This contradictory results may be caused due to our shorter follow-up time. MRI is a more sensitive method comparied with X-ray for determining intervertebral disc degeneration [28]. The shape of adjacent disc may not have changed significantly during the shorter follow-up time.

In control group, the mean VAS score was 7.1±1.8 at the first visit and 5.8±1.4 at the final follow-up, the ODI score improved from 65.7±15.1 to 52.6±13.3 at first visit and final follow-up, respectively. But for the surgical treatment group, the mean VAS score fell dramatically from 7.6±2.2 to 3.2±1.1 immediately after VP and BK procedures. At the final follow-up, the VAS score was 3.5±1.3, but yet statistically nonsignificant, the first day after operation compared with the final follow-up (3.2±1.1 vs. 3.5±1.3, P = 0.147). The ODI score have the same trend as the mean VAS score in the treatment group. Comparison with preoperative and postoperative ODI scores showed improvement in patients underwent VP and BK (68.1±16.2 vs. 36.0±12.4). The final follow-up ODI was 39.5±11.9, which didn't represent a statistically significant difference from the ODI at the evaluation of the first day postoperatively (36.0±12.4 vs. 39.5±11.9, P = 0.759). (Table 4)

**Table 4:** Clinical outcome of all patients at different follow-up time.

		VAS Score			ODI		DHR of the adjacent disc
	1^st^ visit	discharge	2 years	1^st^ visi	discharge	2 years	
Control group	7.1±1.8	6.2±1.6	5.8±1.4	65.7±15.1	62.3±14.7	52.6±13.3	13.5%^c^
Treatment group	7.6±2.2	3.2±1.1^a^	3.5±1.3^a^	68.1±16.2	36.0±12.4^b^	39.5±11.9^b^	17.6%^c^

*P*_a_ = 0.186.

*P*_b_ = 0.127.

*P*_c_ = 0.553.

## Discussion

VP and BK are simple and effective procedures that have been widely accepted as a mini- mally invasive treatment of osteoporotic VCFs. It provides quick pain relief and com- plications are infrequent and mostly minor [29]. Although VP and BK procedures have been shown to be efficacious and obvious for short-term pain relief, it is still controversial whether the two techniques have an impact on the adjacent segments of VCFs by augmentation with bone cement [30], [31], [32]. Previous papers all paid close attention to the incidence of vertebral fracture adjacent to the treated vertebral. However, changes of the adjacent disc after VP and BK procedures has not been involved in previous papers. Our current study aims to assess the degeneration of adjacent disc above the treated vertebral body. We hypothesized VP and BK procedures accelerate the disc degeneration adjacent to the fractured vertebral body.

Previous studies have shown that disc degeneration is influenced by some factors such as, age, body weight and gender [33], [34], [35]. In this paper, in order to increase the reliability of the conclusions, characteristics of patients were also evaluated between two groups. P values of statistical analysis of these factors between the two groups are listed in Table 1. The results showed no significant differences between two groups about these factors. Furthermore, adjacent disc degeneration above the fractured vertebral should not be found on the MRI scan at the first visit. Accordingly, the comparability is reasonable between the two groups. As we all know, age is one of the most important factors of resulting in disc degeneration. For this reason, we only observe the early degeneration of adjacent disc after VP and BK procedures in order to reducing the impact on disc degeneration caused by age.

Our results show that the incidence of adjacent disc degeneration in surgical treatment group is higher than that in control group, and it presents statistically significant difference between the incidence of adjacent disc degeneration in two groups. These suggest VP and BK procedures obviously accelerate the disc degeneration adjacent to the treated vertebral. In addition, we find that a high incidence of disc degeneration occurres in the VP group more than BP group. As a small sample in VP group, we did not conduct statistical analysis between the two groups. Some reasons such as reducing the deformation of the augmented endplate, increasing the pressure in the adjacent intervertebral disc and changing in spinal loading due to cement augmentation are contributed to explain why a higher incidence of adjacent disc degeneration in treatment group. Paul et al. [36] found the deformation of the augmented endplate which was significantly reduced following cement augmentation played an important role in minimizing peak impact loads and reducing strain on intervertebral disc annular fibers. Therefore, the strain on intervertebral disc annular fibers were increased accompany with the reduction of the deformation of the augmented endplate. Applying an axial compression load, Polikeit et al. [37] predicted a pressure increase of 16% above the treated level and 13% below the treated level. Baroud et al. [17] developed a finite-element model to examine cement augmentation on the loading in adjacent vertebral and confirmed the pressure in the adjacent intervertebral disc accordingly increased by approximately 19% and the inward bulge of the endplate adjacent to the one augmented increased considerably, by approximately 17%. Furthermore, Rohlmann et al. [38] found if no compensation of upper body shift was assumed, the force in the erector spine increased by about 200% for the vertebroplasty but by only 55% for the kyphoplasty compared to the intact spine. Intradiscal pressure increased by about 60% and 20% for the vertebroplasty and kyphoplasty, respectively. Rigid cement fixation could theoretically lead to degenerative changes in adjacent segment, and the augmented vertebral is likely much stiffer than the adjacent vertebral [39].

Pain evaluated by VAS score and physical impairment measured by ODI decreased sig- nificantly postoperatively in surgical treatment group. Comparing to control group, VAS score and ODI had mild changes between different follow-up visits. However, statistically significant difference of VAS score and ODI were not found between the first evaluation postoperatively and the final follow-up in the surgical treatment group (P>0.05). This indicates that even if there is a higher incidence of adjacent disc degeneration in the treatment group, but the clinical outcomes have not been weakened because of adjacent disc degeneration.

Some limitations are to be presented in this study. We have a nonrandomized comparative design, and all patients were selected under controlled conditions for a purpose. An unequal number of subjects was classified in two groups and small size of patients was studied, these may lead to biased results. Furthermore, the follow-up period of observing adjacent disc degeneration was relatively short and the adjacent disc degeneration occurred after the follow-up time may be missed.

## Conclusions

In the present study, it suggestes that procedures of VP and BK can accelerate the adjacent disc degeneration above the treated vertebral body. But in the early stage, height reduction of degenerative disc adjacent to the treated vertebral body may not be obvious. Furthermore, clinical outcomes has not been weakened even in the presence of accelerated adjacent disc degeneration.
